# Evaluation of UVC Excimer Lamp (222 nm) Efficacy for Coronavirus Inactivation in an Animal Model

**DOI:** 10.3390/v14092038

**Published:** 2022-09-14

**Authors:** Claudia Maria Tucciarone, Mattia Cecchinato, Lucrezia Vianello, Gabriele Simi, Enrico Borsato, Luca Silvestrin, Michele Giorato, Cristiano Salata, Mauro Morandin, Elisa Greggio, Michele Drigo

**Affiliations:** 1 Department of Animal Medicine, Production and Health (MAPS), University of Padova, Viale dell’Università 16, 35020 Legnaro, Italy; 2 Department of Comparative Biomedicine and Food Science (BCA), University of Padova, Viale dell’Università 16, 35020 Legnaro, Italy; 3 Department of Physics and Astronomy (DFA), University of Padova, Via Marzolo 8, 35131 Padova, Italy; 4 Department of Molecular Medicine (DMM), University of Padova, Via Gabelli 63, 35121 Padova, Italy; 5 National Institute for Nuclear Physics (INFN), Padova Division, Via Marzolo 8, 35131 Padova, Italy; 6 Department of Biology (DiBio), University of Padova, Viale G. Colombo 3, 35131 Padova, Italy

**Keywords:** severe acute respiratory syndrome-related coronavirus-2, infectious bronchitis virus, excimer lamps, UVC, 222 nm, animal model, virus inactivation

## Abstract

The current pandemic caused by severe acute respiratory syndrome-related coronavirus-2 (SARS-CoV-2) has encouraged the evaluation of novel instruments for disinfection and lowering infectious pressure. Ultraviolet subtype C (UVC) excimer lamps with 222 nm wavelength have been tested on airborne pathogens on surfaces and the exposure to this wavelength has been considered safer than conventional UVC. To test the efficacy of UVC excimer lamps on coronaviruses, an animal model mimicking the infection dynamics was implemented. An attenuated vaccine based on infectious bronchitis virus (IBV) was nebulized and irradiated by 222 nm UVC rays before the exposure of a group of day-old chicks to evaluate the virus inactivation. A control group of chicks was exposed to the nebulized vaccine produced in the same conditions but not irradiated by the lamps. The animals of both groups were sampled daily and individually by choanal cleft swabs and tested usign a strain specific real time RT-PCR to evaluate the vaccine replication. Only the birds in the control group were positive, showing an active replication of the vaccine, revealing the efficacy of the lamps in inactivating the vaccine below the infectious dose in the other group.

## 1. Introduction

In December 2019, a new coronavirus that infects humans was discovered [1] and gave origin to a large pandemic that still endures. *Severe acute respiratory syndrome-related coronavirus-2* (SARS-CoV-2) is a *Betacoronavirus* and belongs to the subgenus *Sarbecovirus*, together with SARS-CoV, the virus responsible for Severe acute respiratory syndrome (SARS) [2]. These two viruses are highly contagious airborne pathogens, capable to cause severe pneumonia and systemic disease [3]. However, a fundamental difference allowed SARS-CoV-2 to sustain a tremendous pandemic, which was previously avoided by the containment of SARS-CoV instead [4]. SARS-CoV-2 is in fact shed and transmitted in a preclinical and asymptomatic phase [5], granting viral dissemination by subclinical and unaware hosts travelling [6].

Despite early skepticism, SARS-CoV-2 airborne transmission is now considered pivotal for the virus spread: SARS-CoV-2 is in fact excreted both in droplets and aerosol. Droplets are produced by the upper respiratory tract and possess a diameter wider than 5 µL [7,8], whereas aerosol is constituted by smaller particles and can move further in space. Depending on the particle dimensions, modality of excretion (cough, breath, etc.), and environmental conditions, the virus can deposit swiftly after or it can persist longer in the air [7]. Variations in temperature, humidity, and ozone concentration have shown to affect also SARS-CoV-2 viability [9,10]. Environmental conditions have been thus analyzed to identify critical issues where to intervene. The air diffusion of the virus was proven in many circumstances, such as restaurants, hospitals, public restrooms, and crowded areas [11,12], with the consequent contamination and possible persistence on surfaces [13,14].

SARS-CoV-2 spread has compelled to search for and adopt measures to contain the contagion, such as social distancing, personal protective equipment (PPE), hand sanitization, isolation, quarantine, and disinfection. However, the large-scale implementation of these measures until the pandemic resolution appears greatly expensive, in terms of plastic production, use of disposable materials, and pollution, due to waste and disinfectant release [15,16]. Joining these elements, new solutions are under consideration to lower the environmental infectious pressure of this airborne pathogen.

UVC light application has been considered for SARS-CoV-2 inactivation on cell culture and surfaces [17,18,19], and there is a growing interest towards a particular wavelength (222 nm) produced by excimer lamps that has proven effective for microorganism inactivation [20,21] and especially safe in terms of tissue exposure [22,23,24,25]. However, testing the efficacy of these lamps reproducing in vivo SARS-CoV-2 airborne transmission is not feasible and experimental conditions would require biosecurity level 3 facilities and resources.

For this purpose, the present study evaluated the suitability of an animal model reproducing the airborne transmission of a coronavirus. In fact, coronaviruses are also present in the animal population and cause a wide range of clinical forms, from respiratory to gastroenteric or systemic disease [26,27]. Given the ubiquitous nature of these viruses, their high evolutionary rate, and the overlap of human and animal habitats, spillover phenomena are not infrequent and often cause the pathogen emergence in the human population [28]. Analogies between human and animal coronaviruses can be extended beyond morphology [29], especially in the case of infectious bronchitis virus (IBV) belonging to the species *Avian coronavirus*.

In fact, IBV is a highly contagious respiratory pathogen of chickens that presents a high evolutionary rate and a great number of emerging variants, sometimes escaping immunity [30], similarly to SARS-CoV-2. IBV control largely relies on vaccination; attenuated vaccines are mainly used and administered via spray, they can replicate and sometimes circulate among birds and separate flocks [31]. Due to the value of an animal model for infection dynamics, the use of IBV modified live vaccines has been already proposed to test some biological features of coronaviruses [32], since the vaccine replication after administration to the birds can mimic natural infection.

In the present work, an attenuated IBV vaccine was used on a group of birds to mimic an airborne exposure to a coronavirus in order to verify the functioning of the nebulizing instrument and the efficacy of UVC lamps in inactivating the virus. The residual viability of the virus was also monitored in vivo through time to rule out a delayed replication.

## 2. Materials and Methods

### 2.1. Lamps Description and Aerosolizer Setting

**Figure 1:**
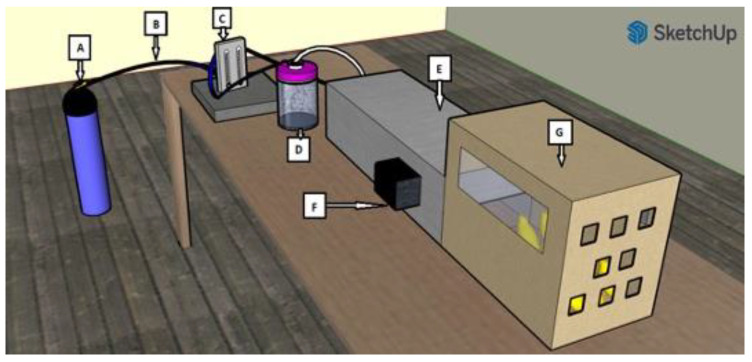
Graphical representation of the settings for nebulization and irradiation of the aerosol: (A) air flow source, (B) T junction, (C) float-type flowmeter, (D) nebulizer, (E) irradiation chamber, (F) UVC (222 nm) lamps, (G) box housing the birds (SketchUp © 2022, Trimble Inc., Sunnyvale, CA, USA).

The instrument containing nebulizer, pump, and lamps (Figure 1) was developed by the Padua Division of the National Institute for Nuclear Physics (INFN) and the Department of Physics and Astronomy (DFA), with minor modifications from Welch et al. (2018) [33]. The air flow was produced by a nitrogen tank connected by a pressure reducer to a T junction, splitting the flow entering a float-type flowmeter. The flow rate was set manually through the flowmeter and a dry part of the flow directly reached the chamber where the lamps were placed; the other part of the flow was directed to the nebulizer (Heart ^®^ Continuous nebulizer, Westmed, Tucson, AZ, USA) containing the virus solution. The particle number and size were measured by a particle sizer PQC 12 EU (PCE Instruments, Italy), being approximately 1 million/m^3^ within 0.3–1 µm, 200,000/m^3^ within 1–2.5 µm, and 10,000/m^3^ more than 2.5 µm. The generated flux was mixed with room air when entering the box containing the birds.

The irradiation chamber was constituted by a 10-cm high, 15-cm wide, and 82-cm-long aluminum tunnel. UVC lamps were placed on the sides of the tunnel, facing one another and four deflectors were added to reproduce the aerosol behavior (Supplementary Figure S1). The lamps were “Care222^®^ filtered far UV-C excimer lamp modules” manufactured by Ushio (Cypress, CA, USA), with a measured total UV power irradiated within an angle of 45°, integrating a value of 50 mW (±10%), and an emission spectrum with a peak at 222 nm (Supplementary Figure S2). Python code [34] was used to develop a simple in house application estimating the average dose delivered to a virus particle by analytically calculating the UV intensity in a mesh of points inside the chamber. This simulation was tuned by measuring the UV intensity in several positions with a photodiode (mod. 918D-UV-OD3, connected to a 1936-C power meter, Newport Corporation, CA, USA). The average dose was calculated by assuming the simple case of a uniform flux of virus particles moving along the chamber at the same constant speed of 1.5 cm/s. The effect of the estimated 20% reflectivity of the aluminum on the average UV intensity was obtained by comparing the measured UV intensity inside the chamber in different directions by means of the photodiode. The effect was found to be of the order of 1% and therefore negligible. The inactivation factor was computed assuming the same sensitivity for this pathogen as for H1N1 in Welch et al. (2018) [33].

At the end of the tunnel, a metal sheet was placed to shield the birds from the UVC lights and avoid direct exposure for safety reasons, since the evaluation of the exposure effect on skin and mucous membranes of superior organisms was not the aim of the present study. The birds were then kept in a container juxtaposed to the end of the tunnel for the duration of the experiment.

### 2.2. Vaccine and Standard Curve

A registered modified live vaccine (Cevac IBird^®^, Ceva Animal Health Ltd., Libourne, France) for infectious bronchitis, based on 1/96 strain, was chosen for the experiment. Two 1000-dose titrated vials (LOT. 028J2S2KGD; 4.2 Log_10_ Embryo Infectious Dose EID_50_/dose), one for each group, were used after resuspension in 100 mL of deionized water. A 200 µL aliquot was processed for nucleic acid extraction with High Pure Viral Nucleic Acid^®^ Kit (Roche, Basel, Switzerland) and used for a standard curve reconstruction by real time RT-PCR following the method published by Tucciarone et al. (2018) [35]. The vaccine was ten-fold diluted and serial dilutions were tested with SuperScript^®^ III Platinum^®^ One-Step qRT-PCR Kit (Invitrogen, Waltham, MA, USA) on LightCycler^®^ 96 Instrument (Roche, Basel, Switzerland).

### 2.3. Animal Model

The present trial was authorized by the Ethics Committee of the University of Padua (Organismo preposto al benessere animale, O.P.B.A.) (protocol code 79/2020, 14 December 2020).

Two groups of 20 commercial broilers of 1 day of age were enrolled in the study 17 days apart to allow a 7-day interval for sanitary measures. Birds were reared in equal environmental conditions for ten days after exposure to the vaccine. Each group was brought from the hatchery to the experimental room where the trial was conducted, before receiving the standard spray vaccination (against multiple pathogens such as infectious bronchitis virus, Newcastle disease virus, etc.) usually administered prior to distribution to farmers, in order to avoid contamination.

The animals were housed in a 2 m × 1.5 m box with litter, infrared lamp, *ad libitum* water, and commercial broiler starter feed, to reproduce farming conditions. At arrival of each group (day 0), 15 chicks out of 20 were individually identified (1–15) by placing a numbered paper string on their legs. The remaining unidentified 5 animals were kept to assure the maximum group size compatible with the box dimension and also ease the attenuated vaccine circulation after replication and shedding. Before exposing the birds to the air flow containing the vaccine, birds from both groups were sampled by choanal cleft swabs; swabs were pooled and tested to detect any preexisting vaccine contamination.

The birds of each group were temporarily housed into a container close to the end of the tunnel, where the UVC lamps were placed, and the vaccine was nebulized into. The first group (group A) was exposed to the air flow containing the vaccine for 40 min and the lamps were turned off to check the correct functioning of the pump system and the viability of the vaccine after the passage through the instrument. During the exposure of the second group (group B), UVC lamps were switched on.

After the exposure, the animals of both groups were moved and housed in the box, where they were fed, watered, clinically inspected, and sampled daily and up to day 9 of the trial, following strict biosecurity measures. Clinical signs were recorded using a scoring system for severity (0 none; 1 mild; 2 moderate; 3 severe) of possible vaccine reaction signs, such as conjunctivitis, nasal discharge, rales, dyspnea, or death [36,37]. Between the housing of the two groups, a careful disinfection was performed, and an empty period of 7 days was observed.

### 2.4. Sample Storage and Laboratory Analyses

Choanal cleft swabs were collected from 15 individually marked birds from day 1 to 9 in each group, in order to obtain longitudinal samples from at least 10 animals in case of possible drop out due to mortality in the first days of life. Swabs were air dried, placed separately into 1.5 mL tubes (identified as follows: group/day/bird), and then stored at −80 °C until the end of the sampling. Samples from the same 10 animals selected at the end of the trial among survivals were processed. The two sample batches were processed separately to avoid contamination and analyses of group A samples were conducted before the beginning of the second trial to evaluate the functioning of the system. Samples were extracted and processed as detailed before and viral titer was calculated using efficiency and one point of the standard curve produced from the vaccine dilutions.

## 3. Results

### 3.1. Lamps Description and Aerosolizer Setting

The settings were regulated to nebulize 10 mL of solution in 40 min of exposure, by supplying 9 L/min of nitrogen and 4.5 L/min of aerosol through a pressurized tank and a 4-bar pressure reducer. The aerosol moved along the chamber at a speed of 1.5 cm/s, granting a UVC exposure of 10 s. A minimal UV irradiation dose of 8.7 mJ/cm^2^ (mean dose 13.6 mJ/cm^2^) and a 10^7^ inactivation factor [33] were estimated.

### 3.2. Vaccine and Standard Curve

The titer of the resuspended vaccine was 158,489 EID_50_/mL (5.2 Log_10_ EID_50_/mL), the limit of detection (LoD) of the real time RT-PCR method used was 10^0.2^ EID_50_/mL, and efficiency was 93%.

### 3.3. Animal Model and Laboratory Analyses

Seven out of 20 birds from group A displayed mild rales intermittently from day 3 to 9, with a peak on days 4 and 9 (4 birds in both days), fully compatible with a mild vaccine reaction. Only one bird in group B displayed mild rales on day 5. There was no evidence of other clinical signs and no birds died during the observation period of both groups.

**Figure 2:**
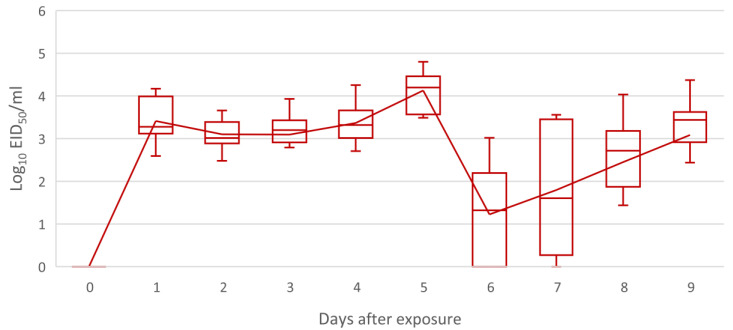
Box plot of vaccine titers (expressed in Log_10_ of EID_50_/mL) of group A; the line represents the mean titer trend.

For each group, 135 individual samples and 1 pool of swabs collected before exposure were obtained. The pre-exposure pooled samples were negative for both groups, excluding a vaccine contamination at the hatchery. Real time RT-PCR results allowed to explore the replication kinetics of the vaccine in group A. All chicks of group A were positive starting from day 1 up to day 5; the few negative results between day 6 and 9 were compatible with intermittent shedding or slight variability in sampling. Only one bird was negative on two consecutive days. Vaccine titers followed similar trends in all birds of group A, where a progressive increase was evident until the peak at day 5 (Figure 2). After the decrease following the peak, a slight second increase in titers appeared in some animals towards day 9 (Figure 2). The minimum and maximum detected vaccine titers were 2.29 (10^0.35^) EID_50_/mL on day 7 and 63,230 (10^4.80^) EID_50_/mL on day 5, respectively. All birds from group B were negative on each sampling point.

## 4. Discussion

The present trial was part of a wider project aiming to evaluate the efficacy of the instrument for the aerosol production and the inactivation capacity of excimer lamps producing 222 nm UVC radiation on coronaviruses. The current pandemic situation has given new impulses to research novel techniques for environmental sterilization, especially against airborne pathogens, and 222 nm wavelength is a promising tool both for its sterilizing activity and lack of hazard for tissues, contrary to other UVC wavelengths [38,39].

The opportunity to use an animal model and a freely manageable virus is unique, since working with in biosafety level 3 conditions for a direct study of coronavirus features can be very hazardous and expensive. Even though conclusions cannot be completely superimposed to other pathogens, a preliminary assessment of the approach robustness and efficacy on shared biological properties is encouraging and time saving. Moreover, the safety of the workers and environment should be safeguarded, and a reduction of risks and exposure should be targeted, when possible.

In this frame, the present study focused on an airborne coronavirus, IBV, which causes disease only in birds, but it shares some important characteristics, both structural and biological, with SARS-CoV-2. IBV immunization in birds is commonly performed by spray vaccination with modified live vaccines, which are taken by the birds through the airways; they replicate and are commonly shed, similarly to natural infection. For this reason, IBV attenuated vaccines were considered suitable to reproduce the airborne transmission of a coronavirus and study the possible residual infectivity, in case low and undetectable amount of virus would have survived the lamp exposure and circulated within the exposed group.

Moreover, IBV vaccine kinetics has been previously described in field and experimental conditions [36,40] that supported the suitability of the group size and trial duration for the vaccine circulation and detection, allowing also to compare the herein obtained results with an expected trend of viral replication.

Given the wide diffusion of IBV vaccination and use of the 1/96-based vaccine, a preliminary screening was essential to rule out any contamination that would have distorted the outcome, and the availability of a quantitative and strain-specific assay [34] for this vaccine helped in this sense. Previous studies [35,36] showed how routine vaccination does not yield an immediate positivity of the animals, since the vaccine needs to start replicating to be detected. However, animals in group A were positive from day 1 and this could be explained by the higher administered dose and size of the inhaled particles. In fact, considering the dilution of the vaccine, the nebulized volume, and duration of the exposure, the birds received a five-times higher dose of vaccine. Moreover, the instrument producing the aerosol was set to reproduce particles of 1–5 µm, which is the most frequently involved size in pathogen transmission among humans [33,41], whereas the normal size of the droplets used in spray vaccination against IBV is around 100–200 µm [31,42]. A larger size normally restricts the penetration of the vaccine to the upper respiratory tract of the birds, preventing a lower replication that could cause more serious adverse reactions. Thus, the higher dose and smaller size of the particles could explain the differences in the vaccine kinetics, such as the absence of a period of negativity, higher titers, and early peak.

Although not in compliance with the routine spray vaccination procedures, both dose and droplet size did not cause any relevant reaction among the birds, except for few episodes of mild rales, that can be considered in line with minor consequences of vaccination and different particle size. Conversely, no clinical signs were evident in group B, where the vaccine was inactivated by the lamps.

The completely different outcome of the trials and the negative results of group B proved the efficacy of the UVC lamps in the inactivation of the virus and the suitability of the animal model to test the correct functioning of the instrument. The length of the trial allowed also to exclude the possibility of a residual viability of the vaccine, which could have replicated among the birds with a certain delay. Even though a direct quantification was not possible in this study, since the measure unit of the vaccine (Embryo Infectious Dose 50) does not indicate the number of virions, and the used diagnostic assay detects also non-viable viral particles, these results can reasonably suggest that the lamps reduced the viral titers below the infectious dose. On the other hand, the variations and increase in titers in group A confirm the viability of the vaccine once passed through the instrument. Further studies must be conducted on the efficacy of the lamps in non-confined environments, where the distance between the UVC-light source and the target of exposure is not controlled.

## 5. Conclusions

This preliminary study shows encouraging results about the virucidal activity of 222 nm-UVC lamps and their use against coronaviruses. These tools could be used for air sanitation in crowded public spaces and in high-risk facilities such as hospitals, especially when conclusive evidence of their safety and standardized exposure protocols will be produced. On the other hand, the efficacy of UVC lamps and new solutions for their application could offer opportunities also in veterinary medicine and in farms, against airborne pathogens such as avian influenza. The implementation of such biosecurity devices will not grant a definitive solution, but they would be useful in the daily fight for lowering the infectious pressure, if proven safe for human exposure by further studies. These instruments, together with other biosecurity measures, such as social distancing and PPE use, and vaccination could help to control this pandemic and be prepared against other future challenges.

## Data Availability

The study did not report any data.

## Supplementary Materials

The following supporting information can be downloaded at: https://www.mdpi.com/article/10.3390/v14092038/s1, Figure S1: Rendering of the aluminum tunnel constituting the irradiation chamber; Figure S2: Emission spectrum of the Ushio lamps showing the 222 nm peak.

## References

[1] Zhou P., Yang X.L., Wang X.G., Hu B., Zhang L., Zhang W., Si H.R., Zhu Y., Li B., Huang C.L. (2020). A pneumonia outbreak associated with a new coronavirus of probable bat origin. Nature.

[2] Gorbalenya A.E., Baker S.C., Baric R.S., de Groot R.J., Drosten C., Gulyaeva A.A., Haagmans B.L., Lauber C., Leontovich A.M., Neuman B.W. (2020). The species Severe acute respiratory syndrome-related coronavirus: Classifying 2019-nCoV and naming it SARS-CoV-2. Nat. Microbiol..

[3] Guan W., Ni Z., Hu Y.Y., Liang W., Ou C., He J., Liu L., Shan H., Lei C., Hui D.S.C. (2020). Clinical Characteristics of Coronavirus Disease 2019 in China. N. Engl. J. Med..

[4] Peiris J.S.M., Chu C.M., Cheng V.C.C., Chan K.H.S., Hung I.F.N., Poon L.L.M., Law K.I., Tang B.S.F., Hon T.Y.W., Chan C.S. (2003). Clinical progression and viral load in a community outbreak of coronavirus-associated SARS pneumonia: A prospective study. Lancet.

[5] To K.K.W., Tsang O.T.Y., Leung W.S., Tam A.R., Wu T.C., Lung D.C., Yip C.C.Y., Cai J.P., Chan J.M.C., Chik T.S.H. (2020). Temporal profiles of viral load in posterior oropharyngeal saliva samples and serum antibody responses during infection by SARS-CoV-2: An observational cohort study. Lancet Infect. Dis..

[6] Li R., Pei S., Chen B., Song Y., Zhang T., Yang W., Shaman J. (2020). Substantial undocumented infection facilitates the rapid dissemination of novel coronavirus (SARS-CoV-2). Science.

[7] Jarvis M.C. (2020). Aerosol Transmission of SARS-CoV-2: Physical Principles and Implications. Front. Public Health.

[8] Tang S., Mao Y., Jones R.M., Tan Q., Ji J.S., Li N., Shen J., Lv Y., Pan L., Ding P. (2020). Aerosol transmission of SARS-CoV-2? Evidence, prevention and control. Environ. Int..

[9] Pyankov O.V., Bodnev S.A., Pyankova O.G., Agranovski I.E. (2018). Survival of aerosolized coronavirus in the ambient air. J. Aerosol. Sci..

[10] Ataei-Pirkooh A., Alavi A., Kianirad M., Bagherzadeh K., Ghasempour A., Pourdakan O., Adl R., Kiani S.J., Mirzaei M., Mehravi B. (2021). Destruction mechanisms of ozone over SARS-CoV-2. Sci. Rep..

[11] Li Y., Qian H., Hang J., Chen X., Cheng P., Ling H., Wang S., Liang P., Li J., Xiao S. (2021). Probable airborne transmission of SARS-CoV-2 in a poorly ventilated restaurant. Build. Environ..

[12] Liu Y.Y., Ning Z., Chen Y., Guo M., Liu Y.Y., Gali N.K., Sun L., Duan Y., Cai J., Westerdahl D. (2020). Aerodynamic analysis of SARS-CoV-2 in two Wuhan hospitals. Nature.

[13] Santarpia J.L., Rivera D.N., Herrera V.L., Morwitzer M.J., Creager H.M., Santarpia G.W., Crown K.K., Brett-Major D.M., Schnaubelt E.R., Broadhurst M.J. (2020). Aerosol and surface contamination of SARS-CoV-2 observed in quarantine and isolation care. Sci. Rep..

[14] Chin A.W.H., Chu J.T.S., Perera M.R.A., Hui K.P.Y., Yen H.-L., Chan M.C.W., Peiris M., Poon L.L.M. (2020). Stability of SARS-CoV-2 in different environmental conditions. Lancet Microbe..

[15] Vanapalli K.R., Sharma H.B., Ranjan V.P., Samal B., Bhattacharya J., Dubey B.K., Goel S. (2021). Challenges and strategies for effective plastic waste management during and post COVID-19 pandemic. Sci. Total Environ..

[16] Murray A.K. (2020). The Novel Coronavirus COVID-19 Outbreak: Global Implications for Antimicrobial Resistance. Front. Microbiol..

[17] Sabino C.P., Sellera F.P., Sales-Medina D.F., Machado R.R.G., Durigon E.L., Freitas-Junior L.H., Ribeiro M.S. (2020). UV-C (254 nm) lethal doses for SARS-CoV-2. Photodiagnosis Photodyn. Ther..

[18] Kitagawa H., Nomura T., Nazmul T., Omori K., Shigemoto N., Sakaguchi T., Ohge H. (2021). Effectiveness of 222-nm ultraviolet light on disinfecting SARS-CoV-2 surface contamination. Am. J. Infect. Control.

[19] Storm N., McKay L.G.A., Downs S.N., Johnson R.I., Birru D., de Samber M., Willaert W., Cennini G., Griffiths A. (2020). Rapid and complete inactivation of SARS-CoV-2 by ultraviolet-C irradiation. Sci. Rep..

[20] Eadie E., Hiwar W., Fletcher L., Tidswell E., O’Mahoney P., Buonanno M., Welch D., Adamson C.S., Brenner D.J., Noakes C. (2022). Far-UVC (222 nm) efficiently inactivates an airborne pathogen in a room-sized chamber. Sci. Rep..

[21] Buonanno M., Welch D., Shuryak I., Brenner D.J. (2020). Far-UVC light (222 nm) efficiently and safely inactivates airborne human coronaviruses. Sci. Rep..

[22] Buonanno M., Ponnaiya B., Welch D., Stanislauskas M., Randers-Pehrson G., Smilenov L., Lowy F.D., Owens D.M., Brenner D.J. (2017). Germicidal efficacy and mammalian skin safety of 222-nm UV light. Radiat. Res..

[23] Yamano N., Kunisada M., Kaidzu S., Sugihara K., Nishiaki-Sawada A., Ohashi H., Yoshioka A., Igarashi T., Ohira A., Tanito M. (2020). Long-term Effects of 222-nm ultraviolet radiation C Sterilizing Lamps on Mice Susceptible to Ultraviolet Radiation. Photochem. Photobiol..

[24] Fukui T., Niikura T., Oda T., Kumabe Y., Ohashi H., Sasaki M., Igarashi T., Kunisada M., Yamano N., Oe K. (2020). Exploratory clinical trial on the safety and bactericidal effect of 222-nm ultraviolet C irradiation in healthy humans. PLoS ONE.

[25] Narita K., Asano K., Morimoto Y., Igarashi T., Nakane A. (2018). Chronic irradiation with 222-nm UVC light induces neither DNA damage nor epidermal lesions in mouse skin, even at high doses. PLoS ONE.

[26] Zappulli V., Ferro S., Bonsembiante F., Brocca G., Calore A., Cavicchioli L., Centelleghe C., Corazzola G., De Vreese S., Gelain M.E. (2020). Pathology of Coronavirus Infections: A Review of Lesions in Animals in the One-Health Perspective. Animals.

[27] Decaro N., Lorusso A. (2020). Novel human coronavirus (SARS-CoV-2): A lesson from animal coronaviruses. Vet. Microbiol..

[28] Ruiz-Aravena M., McKee C., Gamble A., Lunn T., Morris A., Snedden C.E., Yinda C.K., Port J.R., Buchholz D.W., Yeo Y.Y. (2022). Ecology, evolution and spillover of coronaviruses from bats. Nat. Rev. Microbiol..

[29] Marty A.M., Jones M.K. (2020). The novel Coronavirus (SARS-CoV-2) is a one health issue. One Health.

[30] Jackwood M.W., Wit S., De Wit S. (2020). Infectious Bronchitis. Diseases of Poultry.

[31] Jordan B. (2017). Vaccination against infectious bronchitis virus: A continuous challenge. Vet. Microbiol..

[32] Nefedova E., Koptev V., Bobikova A.S., Cherepushkina V., Mironova T., Afonyushkin V., Shkil N., Donchenko N., Kozlova Y., Sigareva N. (2021). The Infectious Bronchitis Coronavirus Pneumonia Model Presenting a Novel Insight for the SARS-CoV-2 Dissemination Route. Vet. Sci..

[33] Welch D., Buonanno M., Grilj V., Shuryak I., Crickmore C., Bigelow A.W., Randers-Pehrson G., Johnson G.W., Brenner D.J. (2018). Far-UVC light: A new tool to control the spread of airborne-mediated microbial diseases. Sci. Rep..

[34] Van Rossum G., Drake F.L. (2009). Python 3 Reference Manual.

[35] Tucciarone C.M., Franzo G., Berto G., Drigo M., Ramon G., Koutoulis K.C., Catelli E., Cecchinato M. (2018). Evaluation of 793/B-like and Mass-like vaccine strain kinetics in experimental and field conditions by real-Time RT-PCR quantification. Poult. Sci..

[36] Tucciarone C.M., Franzo G., Bianco A., Berto G., Ramon G., Paulet P., Koutoulis K.C., Cecchinato M. (2018). Infectious bronchitis virus gel vaccination: Evaluation of Mass-like (B-48) and 793/B-like (1/96) vaccine kinetics after combined administration at 1 day of age. Poult. Sci..

[37] Jackwood M.W., Rosenbloom R., Petteruti M., Hilt D.A., McCall A.W., Williams S.M. (2010). Avian coronavirus infectious bronchitis virus susceptibility to botanical oleoresins and essential oils in vitro and in vivo. Virus Res..

[38] Pfeifer G.P., Besaratinia A. (2012). UV wavelength-dependent DNA damage and human non-melanoma and melanoma skin cancer. Photochem. Photobiol. Sci..

[39] Pfeifer G.P., You Y.H., Besaratinia A. (2005). Mutations induced by ultraviolet light. Mutat. Res.—Fundam. Mol. Mech. Mutagen..

[40] Pellattiero E., Tucciarone C.M., Franzo G., Berto G., Koutoulis K., Meini A., Zangrandi C., Ramon G., Drigo M., Cecchinato M. (2018). Evaluation of unintended 1/96 infectious bronchitis vaccine transmission in broilers after direct contact with vaccinated ones. Vet. Med..

[41] Papineni R.S., Rosenthal F.S. (1997). The size distribution of droplets in the exhaled breath of healthy human subjects. J. Aerosol Med. Depos. Clear. Eff. Lung.

[42] Lim T.H., Youn H.N., Yuk S.S., Kwon J.H., Hong W.T., Bin Gwon G., Lee J.A., Lee J.B., Lee S.W., Song C.S. (2015). Successful cross-protective efficacy induced by heat-adapted live attenuated nephropathogenic infectious bronchitis virus derived from a natural recombinant strain. Vaccine.

