# Supplementary material for: Evaluation of UVC Excimer Lamp (222 nm) Efficacy for Coronavirus Inactivation in an Animal Model
Source: Viruses. 2022 Sep 14;14(9):2038. doi: 10.3390/v14092038 (PMC9503014; doi:10.3390/v14092038)
Supplement: Supplementary file 1 [file viruses-14-02038-s001.zip › viruses-1878395-supplementary.pdf]

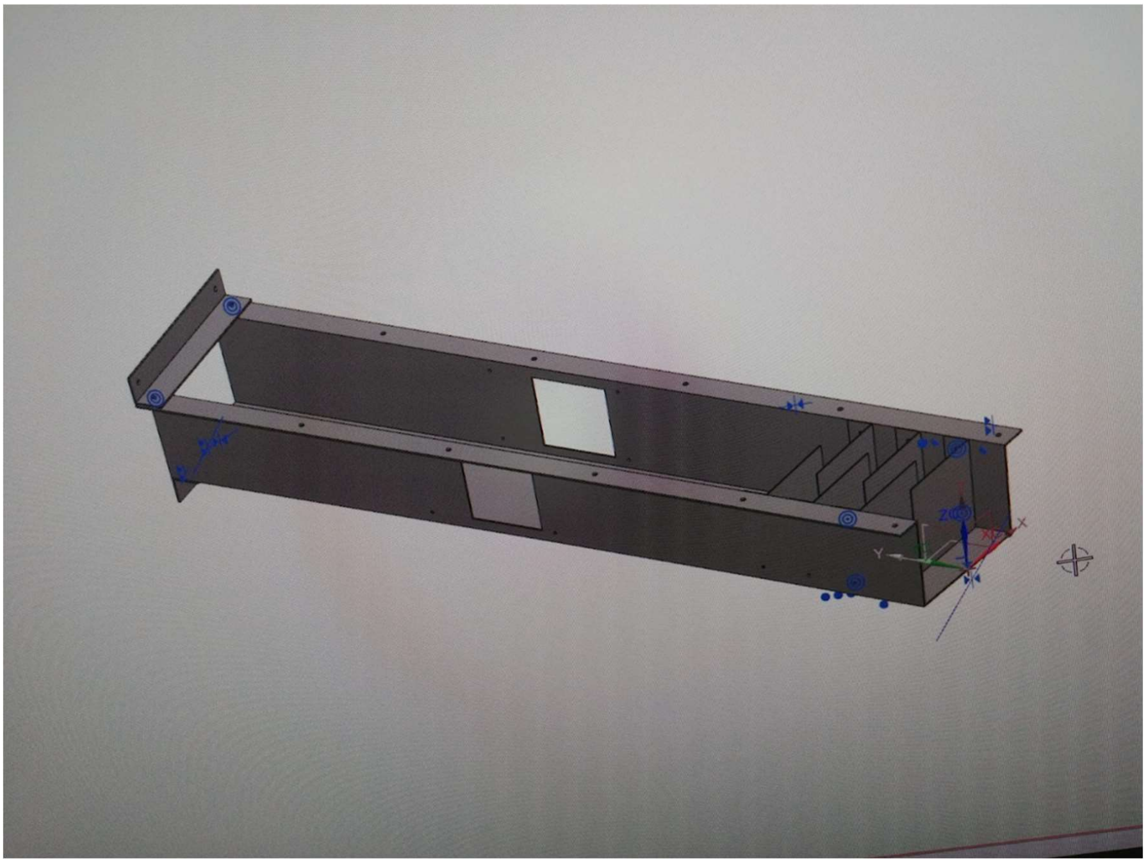

Figure S1. Rendering of the aluminum tunnel constituting the irradiation chamber.

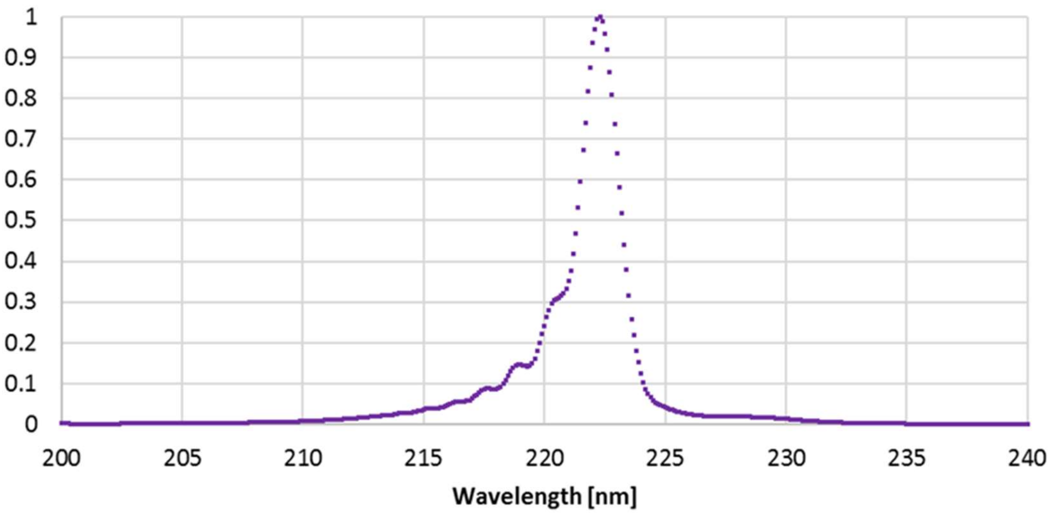

Figure S2. Emission spectrum of the Ushio lamps showing the 222 nm peak.
